# Emotionale Belastung von Medizinstudierenden im Rahmen der universitären Lehre zu psychischen Erkrankungen

**DOI:** 10.1007/s00115-024-01774-7

**Published:** 2024-11-11

**Authors:** Matthias Besse, Michael Belz

**Affiliations:** Klinik für Psychiatrie und Psychotherapie, Universitätsmedizin Göttingen, Von Siebold-Str. 5, 37075 Göttingen, Deutschland

**Keywords:** Depression, Stress, Mentale Gesundheit, Psychiatrische Lehre, Medizinstudium, Stress, Depression, Mental health, Psychiatric teaching, Medical studies

## Abstract

**Hintergrund:**

Zahlreiche Studien belegen eine hohe Ausprägung sowie Zunahme stressassoziierter Symptome und depressionsähnlicher Beschwerden bei Studierenden. Im Rahmen der Lehre zu psychischen Erkrankungen an unserer medizinischen Fakultät berichteten Studierende wiederholt emotional belastende Situationen und äußerten den Wunsch nach Unterstützungsangeboten. Ziel der vorliegenden Studie war die Objektivierung des Belastungslevels und des Bedarfs nach Unterstützungsangeboten.

**Material und Methoden:**

Insgesamt 118 Studierende des 9. Semesters (WiSe 22/23) wurden mittels Fragebogen zu ihrer emotionalen Belastung befragt. Der Fragebogen wurde als Onlinebefragung in LimeSurvey (LimeSurvey GmbH, Hamburg, Deutschland) durchgeführt und enthielt insgesamt 18 als Statements formulierte sowie weitere Items. Zu den einzelnen Items konnten die Studierenden auf 11-stufigen numerischen Skalen mit Außenankern Ratings abgeben.

**Ergebnisse:**

Die eigene Belastung im Vergleich zu anderen Modulen wurde als erhöht eingeschätzt – insbesondere für das Fach Psychiatrie (> 5 von 10). Am belastendsten wurde der Unterricht am Krankenbett, gefolgt von Seminaren und Vorlesungen eingeschätzt (alle Paarvergleiche *p* < 0,001). Inhalte zu Depression erzeugten überwiegend die stärkste Belastung. Etwa die Hälfte der Studierenden wünschte sich unterstützende Angebote; hiervon am häufigsten eine Sprechstunde (78,0 %) sowie feste Ansprechpartner (70,7 %).

**Diskussion:**

Die emotionale Belastung von Studierenden im Rahmen der Lehre zu psychischen Erkrankungen ist insbesondere im Fach Psychiatrie sowie mit ansteigendem Patientenkontakt höher, Unterstützungsangebote werden gefordert. Lehrende sollten hierzu sensibilisiert werden und Angebote wie offene Sprechstunden oder die Benennung fester Ansprechpartner schaffen.

## Abstract (en)

**Background:**

Numerous studies have shown a high incidence and increase in stress-related symptoms and depression-like complaints among students. During teaching on mental disorders at our medical faculty, students repeatedly reported emotionally stressful situations and expressed a desire for support services. The aim of the present study was to objectify the level of stress and the need for such support services.

**Material and methods:**

A total of 118 students in the 9th semester (winter semester 2022/2023) were surveyed using a questionnaire on their emotional stress. The online questionnaire was conducted via LimeSurvey (LimeSurvey GmbH, Hamburg, Germany) and contained a total of 18 items formulated as statements as well as other items. The students rated the individual items on 11-point numerical scales with external anchor ratings.

**Results:**

The respondents rated their own stress as increased compared to other modules, particularly for the subject of psychiatry (> 5 out of 10). Bedside teaching was estimated as being the most stressful, followed by seminars and lectures (all pairwise comparisons *p* < 0.001). The contents on depression predominantly caused the most stress. Approximately half of the students would like to be offered support services and most frequently a consultation session (78.0%) and defined contact persons (70.7%).

**Conclusion:**

The emotional stress of students in the context of teaching on mental disorders is particularly high in psychiatry and higher with increasing patient contact; offers of support services are needed. Teachers should be sensitized to this and create offers such as open consultation sessions and the provision of defined contact persons.

## Hintergrund und Fragestellung

In einer 2023 von der Techniker Krankenkasse in Auftrag gegebenen Umfrage wurden 1000 repräsentativ ausgewählte Studierende zu gesundheitsbezogenen Themen querschnittlich befragt. Zentrales Ergebnis dieser Studie war, dass ein großer Teil der deutschen Studierenden über Erschöpfungssymptome (68 %), unspezifische Schmerzen (59 %) und depressive Verstimmung (34 %) klagte. Zudem bezeichneten ca. 10 % (vgl. 2015: 3 %) ihre Gesundheit als „weniger gut“ oder „schlecht“ [17]. Generell sind psychische Erkrankungen mit über 65 % der häufigste Grund für studienerschwerende Beeinträchtigungen und liegen bei etwa 16 % der Studierenden vor [7].

Mit einer Regelstudienzeit von 12 Semestern zählt das Medizinstudium zu den längsten Studiengängen [9]. Obschon die Abbruchquote mit ca. 11 % relativ niedrig ist [15], wird dem Medizinstudium ein hoher Schweregrad zugesprochen. Vor Aufnahme des Studiums müssen sich Interessierte mit exzellenten Abiturnoten oder in Auswahlverfahren an den Hochschulen beweisen, um die Chance auf einen Studienplatz zu erhalten [10]. Etwa 16 % der Medizinstudierenden weisen schon kurz vor Aufnahme ihres Studiums leichte depressive Symptome auf, wobei sich der Anteil im 1. Semester auf über 21 % erhöht [12]. Auch in späteren Semestern persistieren hohe Werte für Depressivität und Stresserleben [16].

An unserer medizinischen Fakultät wurde im Rahmen der studentischen Evaluation zum Modul „Nervensystem und Psyche“, in welchem die Lehre zu psychiatrischen und psychosomatischen Krankheitsbildern stattfindet, wiederholt in Freitextantworten von emotional belastenden Situationen berichtet. Vereinzelt gaben Studierende an, selbst an einer psychischen Erkrankung zu leiden und sich hierdurch vulnerabel gegenüber den Modulinhalten zu fühlen. Der Wunsch nach Unterstützungsangeboten wurde wiederholt mündlich und per Mail an die Modulkoordination herangetragen. Persönliche Gespräche im Anschluss an Lehrveranstaltungen bestätigten diesen Eindruck.

Bislang gibt es keine Veröffentlichungen zur emotionalen Belastung von Medizinstudierenden während der Lehre zu psychischen Krankheitsbildern. Die vorliegende Studie soll diese Lücke schließen und das Belastungslevel objektivieren. An der Universitätsmedizin Göttingen (UMG) wurde eine Befragung der Studierenden zur emotionalen Belastung durch unterschiedliche Lehrinhalte im Modul „Nervensystem und Psyche“ durchgeführt.

Die primäre Zielsetzung der Studie ist die Abfrage und Objektivierung des Belastungslevels der Studierenden (1a) generell im Umgang mit psychischen Krankheitsbildern, (1b) zwischen den Fächern des Moduls (Psychiatrie, Kinder- und Jugendpsychiatrie sowie psychosomatische Medizin) sowie (1c) bezogen auf die Lehrform und damit verbundene Situationen, in denen psychische Krankheitsbilder präsentiert werden (Vorlesung, Seminar, UaK). Weiterhin ist es primäres Ziel der Studie, (2) das Belastungslevel in Abhängigkeit von neun ausgewählten psychischen Krankheitsbildern zu erfassen (u. a. Depression, Demenz). Zuletzt soll (3) der Bedarf nach Unterstützungsangeboten objektiviert werden.

## Methodik

### Stichprobe und Studiendesign

Es wurde eine Onlinebefragung einmalig und zur Minimierung eines Rückschaufehlers unmittelbar nach Abschluss des Moduls 5.1 im Rahmen des klinischen Studienabschnitts Medizin (s. Abschn.: Beschreibung der Lehre: Modul 5.1) mit LimeSurvey durchgeführt – Teilnahmelinks wurden innerhalb von 24 h nach dem letzten UaK per E‑Mail an die Studierenden verschickt, Antworten gingen vom 22.11.2022 bis zum 18.12.2022 ein. Von 118 Studierenden im 9. Semester (WiSe22/23) nahmen *N* = 83 teil (Rücklaufquote: 70,3 %). Zur Wahrung der Anonymität wurden keine demographischen Daten erhoben (Geschlecht, Alter). Freitextantworten wurden vor Auswertung auf Inhalte geprüft, die zu einer Identifizierung von Einzelpersonen geeignet gewesen wären. Eine Zustimmung der Ethikkommission der UMG wurde eingeholt (AZ 26/6/22).

### Beschreibung der Lehre: Modul 5.1

An unserer Universität ist der Studiengang Humanmedizin als klassischer Regelstudiengang mit vier vorklinischen und sechs klinischen Semestern sowie dem Praktischen Jahr organisiert. Die klinischen Fächer werden in interdisziplinär besetzen Modulen mit meist 3‑ bis 7‑wöchigem Umfang unterrichtet. Im Modul 5.1 („Nervensystem und Psyche“) des 9. Semesters findet die interdisziplinäre Lehre zu psychiatrischen, kinder- und jugendpsychiatrischen sowie psychosomatischen Krankheitsbildern („Psych. Block“) statt. Zudem werden Erkrankungen aus dem Bereich der Neurologie unterrichtet.

Im WiSe22/23 wurden die theoretischen Grundlagen zu den Krankheitsbildern über Vorlesungen und im Fach Psychiatrie zusätzlich in drei 90-minütigen interaktiven Kleingruppenseminaren vermittelt. Hierbei wurden Patienten in das Seminar mit einbezogen. Im sich über zwei volle Tage erstreckenden UaK wendeten die Studierenden ihr erworbenes theoretisches Wissen an und explorierten selbstständig im direkten Kontakt bei mehreren Patienten die Symptome der verschiedenen psychischen Krankheitsbilder (in der Regel Demenz, Schizophrenie, affektive Störungen, Persönlichkeitsstörungen, Suchterkrankungen, posttraumatische Belastungsstörungen, Angst- und Zwangserkrankungen). Die Modulklausur wurde nach den theoretischen Lehrveranstaltungen und unmittelbar vor dem Block mit UaK geschrieben.

### Fragebogen zur emotionalen Belastung von Medizinstudierenden

Der von den Autoren entworfene Fragebogen erfasste die Teilnahme an den Veranstaltungen im Modul 5.1 („Vorlesung“; „Seminar“; „UaK“). Im Folgenden wurden den Studierenden 18 Items als Statements präsentiert, zu denen auf 11-stufigen numerischen Skalen mit Außenankern Ratings abgegeben werden konnten (0 = „trifft überhaupt nicht zu“; 10 = „trifft voll zu“; s. Tab. 1 für eine detaillierte Übersicht). Block 1 (Items 1.1 bis 3.3) erfasste die generelle *Belastung* durch Lehre mit Bezug zu psychischen Krankheitsbildern. Block 2 (Items 4.1 bis 4.9) erfasste die spezifische Belastung durch neun ausgewählte *Krankheitsbilder*.

**Tab. 1:** Formulierung der Fragebogenitems

Itemformulierungen	Antwortskala
**Block 1: Belastungen**	
1.1 „Der Psych.-Block des Moduls 5.1 hat mich stärker emotional belastet als andere Module des klinischen Studienabschnitts.“	11-stufig^a^
1.2 „Ich kenne viele andere Studierende, die den Psych.-Block des Moduls 5.1 emotional belastend fanden.“	
1.3 „Mir persönlich fällt der Umgang mit psychischen Erkrankungen generell schwerer als mit somatischen Erkrankungen.“	
*2. „Von den Fächern des Psych.-Blocks hat mich das folgende am meisten emotional belastet:“*	
2.1 „Psychiatrie“	11-stufig^a^
2.2 „Kinder- und Jugendpsychiatrie“	
2.3 „Psychosomatische Medizin“	
*3. „Folgende Situationen*^b^ *habe ich als besonders emotional belastend für ich erlebt:“*	
3.1 „Theoretische Beschäftigung mit den psychischen Krankheitsbildern“	11-stufig^a^
3.2 „Direkter Kontakt zu psychisch kranken Menschen im Seminar“	
3.3 „Direkter Kontakt zu psychisch kranken Menschen im UaK“	
**Block 2: Krankheitsbilder**	
*4. „Die Beschäftigung mit folgenden Krankheitsbildern*^*b*^^, c^ *hat mich emotional besonders belastet:“*	
4.1–4.9 „Demenzen und andere organische Störungen“; […]; „Persönlichkeitsstörungen“	11-stufig^a^
**Block 3: Wunsch nach unterstützenden Angeboten**	
5. „Ich hätte mir Hinweise auf eine Anlaufstelle gewünscht, an die ich mich aufgrund der emotionalen Belastung im Modul 5.1 hätte wenden können:“	„Ja“ vs. „Nein“
*6*^*b,*^ ^*d*^*. „Ich hätte mir die folgenden Angebote/Hinweise auf bestehende Angebote gewünscht:“*	
6.1 „Psychosoziale Ambulanz für Studierende“	„Ja“ vs. „nicht gewählt“
6.2 „Vorwarnung bei möglicherweise belastenden Themen“	
6.3 „Feste*r Ansprechpartner*in für emotional belastete Studierende“	
6.4 „Offene Sprechstunde für Studierende“	

Alle Statements (Items Nr. 1 bis 4) konnten auf einer ^a^11-stufigen numerischen Skala mit zwei Ankern von 0 = „trifft überhaupt nicht zu“ bis 10 = „trifft voll zu“ beantwortet werden. In den Items Nr. 3, 4 und 6 konnten ^b^„*sonstige* Situationen“, ^b^„*sonstige* Krankheitsbilder“ sowie ^b^„*sonstige* Angebote“ im Freitext geschildert werden. Für Item Nr. 4 wurden den Studierenden ^c^9 unterschiedliche Krankheitsbilder zur Einstufung der emotionalen Belastung präsentiert; siehe Abb. 2. ^d^Item Nr. 6 wurde nur bei der Antwort „Ja“ in Item Nr. 5 präsentiert

Im Block 3 wurde der *Wunsch nach unterstützenden Angeboten *erfasst: Zum einen der Wunsch nach einer Anlaufstelle (Item 5; „Ja“ vs. „Nein“). Die mit „Ja“ antwortenden Studierenden konnten anschließend spezifizieren, welche Hinweise auf bestehende bzw. neue Angebote sie sich gewünscht hätten (Item 6; s. Tab. 1).

Zum anderen wurden die Studierenden zusätzlich gefragt, ob sie sich aktuell „[…] selbst aufgrund einer psychischen Erkrankung in Behandlung“ befanden („Ja“ vs. „Nein“ vs. „keine Angabe“) und ob sich die Symptome dieser Erkrankung im Rahmen des „Psych.-Blocks“ verschlechtert hätten (Rating: 0 = „trifft überhaupt nicht zu“; 10 = „trifft voll zu“).

### Statistische Auswertung

Die Analyse der Daten erfolgte mit SPSS®, Version 29 (IBM, Armonk, NY, USA). Für die deskriptive Darstellung einzelner Items wurden Häufigkeiten, Mittelwerte (M), Standardabweichungen (SD) und Pearson-Korrelationen berechnet (r). Zudem wurde für die numerischen Ratings die Häufigkeit der Studierenden berechnet, die ein als „hoch“ definiertes Belastungsniveau angaben (Werte von 8–10, s. Abbildungen). Freitextantworten wurden nur bei Abweichung von denen im Fragebogen erfassten Informationen kategorisiert und berichtet.

Die Analyse der primären Endpunkte in den Blöcken 1 (*Belastungen*) und 2 (*Krankheitsbilder*) erfolgte mit multiplen allgemeinen linearen Modellen (GLM) für messwiederholte Daten – hiervon ausgenommen waren die Items 1.1 bis 1.3, die lediglich deskriptiv ausgewertet wurden. Die Ratings wurden als Innersubjektfaktor in das jeweilige Modell integriert. Es wurden insgesamt drei GLM erstellt (Block 1: Itemgruppen 2 und 3; Block 2: Itemgruppe 4; s. Tab. 1 für alle Items). Für jedes GLM wurde der Innersubjekteffekt auf Signifikanz getestet und zudem Bonferroni-korrigierte paarweise Vergleiche auf Itemebene durchgeführt. Für den Block 3 (*Wunsch nach unterstützenden Angeboten*) wurde eine deskriptive Auswertung durchgeführt (Häufigkeiten bzw. Prozentwerte). Das initiale Signifikanzniveau wurde festgelegt auf α < 0,05 (zweiseitig). Die Studierenden konnten einzelne Items im Fragebogen auslassen; für die jeweiligen GLM werden die gültigen Fälle (listenweise) im Ergebnisteil angegeben.

Die Auswertung der Angaben zu eigener psychischer Erkrankung fand explorativ statt (Details s. Abschn.: Hinweise auf erhöhte Vulnerabilität bei psychisch vorerkrankten Studierenden).

## Ergebnisse

### Vollständigkeit der Befragung und Teilnahme an der Lehre

Von *n* = 78 (94,0 %) Studierenden wurden alle Ratings (Block 1 und 2; 18 Items) vollständig ausgefüllt; *n* = 5 ließen je ein einzelnes Item aus. Es besuchten *n* = 62 (74,7 %) die Vorlesung, jeweils *n* = 79 (95,2 %) Seminar und UaK.

### Block 1: Hohe Belastung durch psychiatrische Lehre und Patientenkontakt

**Abb. 1:**
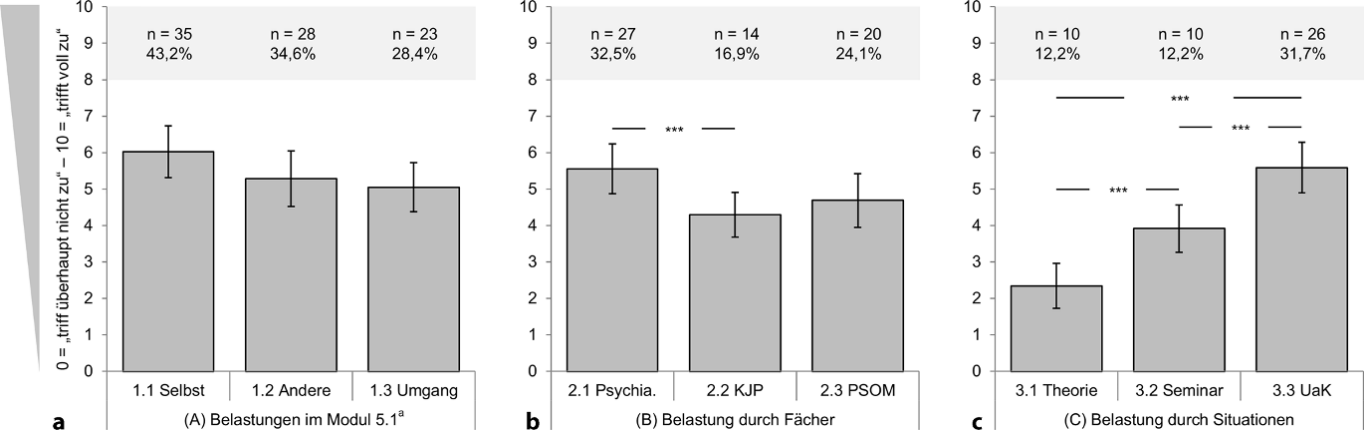
Einschätzung von **a** emotionaler *Belastung* im Modul 5.1 (Items 1.1 bis 1.3; *N* = 81, ^a^ausschließlich deskriptive Auswertung), **b** Belastung durch spezifische *Fächer* (Items 2.1 bis 2.3; *N* = 83) und **c** Belastung durch spezifische *Situationen* (Patientenkontakt; Items 3.1 bis 3.3; *N* = 82). Mittelwerte mit 95 %-Konfidenzintervallen und Bonferroni-korrigierten Paarvergleichen (GLM); Häufigkeiten des Belastungsniveaus „hoch“ (Werte 8–10 von 10). Für alle Itemformulierungen s. Tab. 1. ** *p* < 0,01; *** *p* < 0,001

Die Studierenden gaben für die Items 1.1 bis 1.3 (generelle Belastung) im Mittel ausschließlich Ratings über dem Skalenmittelwert von „5“ an (Abb. 1a). Die „erhöhte eigene emotionale Belastung im Vergleich zu anderen Modulen des klinischen Studienabschnitts“ (Item 1.1) war am höchsten ausgeprägt (M = 6,02, SD = 3,25), gefolgt von der Einschätzung, „auch andere Studierende seien hierdurch stärker belastet“ (Item 1.2; M = 5,28, SD = 3,5) und der Aussage, dass der „Umgang mit psychischen Erkrankungen generell schwerer falle als der Umgang mit somatischen Erkrankungen“ (Item 1.3; M = 5,05, SD = 3,10).

Bezogen auf die Fächer des Moduls (Items 2.1 bis 2.3, Abb. 1b) empfanden die Studierenden „Psychiatrie“ als am stärksten belastend (M = 5,55, SD = 3,16), gefolgt von „psychosomatischer Medizin“ (M = 4,69, SD = 3,43) und „Kinder- und Jugendpsychiatrie“ (M = 4,29, SD = 2,84). Erneut konnte ein signifikanter Innersubjekteffekt gefunden werden (GLM: *N* = 83 gültige Fälle; F [2, 164] = 7,56, *p* < 0,001, partielles η^2^ = 0,08): Die Belastung im Fach „Psychiatrie“ war signifikant höher als die Belastung im Fach „Kinder- und Jugendpsychiatrie“ (*p* < 0,001; alle weiteren Paarvergleiche n.s.).

Als am stärksten emotional belastende Situation (Items 3.1 bis 3.3; Abb. 1c) wurde der UaK bewertet (M = 5,59, SD = 3,21), gefolgt von Seminar (M = 3,91, SD = 3,00) und Vorlesung (M = 2,34, SD = 2,86). Es konnte ein signifikanter Innersubjekteffekt gefunden werden (GLM: *N* = 82 gültige Fälle; F [2, 162] = 45,18, *p* < 0,001, partielles η^2^ = 0,36); die Unterschiede zwischen allen drei Lehrformen waren signifikant (alle Paarvergleiche *p* < 0,001).

Freitextantworten zu besonders emotional belastenden Situationen wurden von *n* = 18 (21,7 %) abgegeben. Am häufigsten wurde Belastung durch von Patienten geschilderte „Schicksalsschläge“ (*n* = 6) und Belastung durch Identifikation mit Patienten in ähnlichem Alter/Erkrankungen wie die Studierenden (*n* = 2) genannt.

### Block 2: Depression und PTBS als emotional belastende Krankheitsbilder

**Abb. 2:**
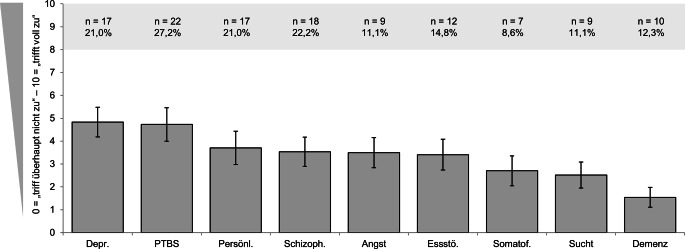
Einschätzung emotionaler Belastung durch unterschiedliche psychische Krankheitsbilder (Items 4.1 bis 4.9). Mittelwerte mit 95 %-Konfidenzintervallen; Häufigkeiten des Belastungsniveaus „hoch“ (Werte von 8–10 von 10). Für alle Itemformulierungen s. Tab. 1. *N* = 81. *PTBS* posttraumatische Belastungsstörung

Die stärkste Belastung (Abb. 2 für eine Übersicht) wurde für „Depression“ angegeben (M = 4,83, SD = 2,97), gefolgt von „posttraumatischer Belastungsstörung“ (PTBS; M = 4,73, SD = 3,37). Die geringste Belastung gaben die Studierenden für „Demenzen und andere organische Störung“ an (M = 1,54, SD = 1,99). Der Innersubjekteffekt war signifikant (GLM: *N* = 81 gültige Fälle; F [8, 640] = 18,97, *p* < 0,001, partielles η^2^ = 0,19). Von 36 Paarvergleichen erreichten 17 die Signifikanz. Maßgeblich bildeten diese die im Vergleich zu allen restlichen Störungsbildern signifikant erhöhte Belastung durch den Bereich „Depression“ ab (*p* = 0,044 bis < 0,001; mit Ausnahme von „PTBS“: *p* = 0,99). „Demenzen und andere organische Störung“ erzeugte bei den Studierenden eine signifikant niedrigere Belastung als die restlichen Störungsbilder (*p* = 0,033 bis < 0,001; mit Ausnahme der „Suchterkrankungen“: *p* = 0,078).

### Block 3: Großer Wunsch nach Unterstützungsangeboten

**Abb. 3:**
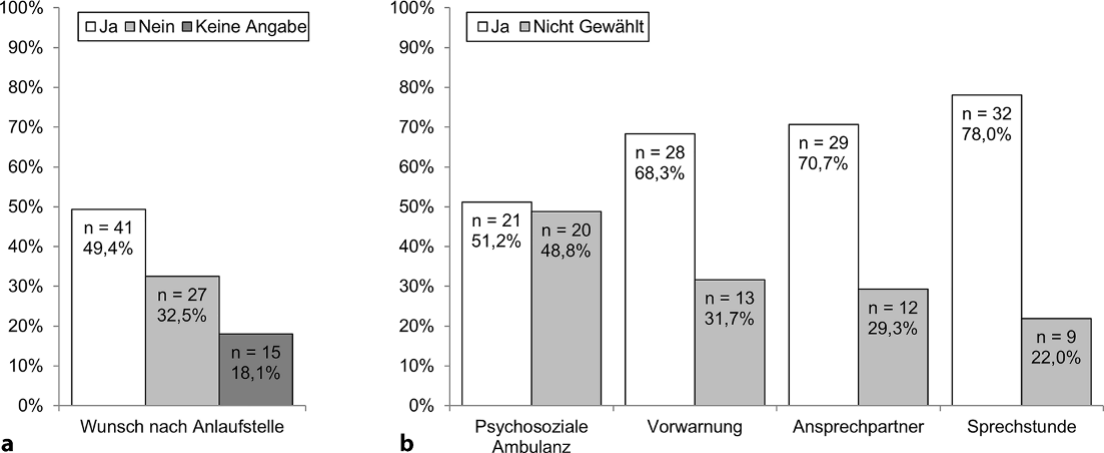
Häufigkeiten und Prozentwerte zu **a** dem Wunsch nach einer *Anlaufstelle* aufgrund der emotionalen Belastung in Modul 5.1 (Item 5; *N* = 83) und **b** konkreten *Unterstützungsangeboten* bzw. Hinweis auf diese (Items 6.1 bis 6.4; *n* = 41). Für alle Itemformulierungen s. Tab. 1

Es gaben *n* = 41 (49,4 %) der Studierenden an, sich „Hinweise auf eine Anlaufstelle“ aufgrund der emotionalen Belastung im Modul gewünscht zu haben („nein“: *n* = 27, 32,5 %; „keine Angabe“: *n* = 15, 18,1 %; Abb. 3a).

Von den *n* = 41 Studierenden gaben *n* = 21 (51,2 %) den Hinweis auf die bestehende „Psychosoziale Ambulanz für Studierende (PAS)“ als Wunsch an. Eine „Vorwarnung bei möglicherweise belastenden Themen“ wünschten sich *n* = 28 (68,3 %), „eine/n feste/n Ansprechpartner/in“ *n* = 29 (70,7 %). Der stärkste Wunsch bestand nach einer „offenen Sprechstunde für Studierende“ (*n* = 32, 78,0 %; Items 7.1 bis 7.4, Abb. 3b).

### Hinweise auf erhöhte Vulnerabilität bei psychisch vorerkrankten Studierenden

Es gaben *n* = 15 (18,1 %) an, aufgrund einer psychischen Erkrankung behandelt zu werden („nein“: *n* = 64, 77,1 %; „keine Angabe“: *n* = 4, 4,8 %). Diese Studierenden in Behandlung bewerteten ein Statement mittels 11-stufiger Skala („Symptome meiner psychischen Erkrankung haben sich im Rahmen des Psych.-Blocks im Modul 5.1 vorübergehend verschlechtert“) mit M = 4,67 (SD = 3,02).

Explorativ wurde analysiert, ob der Status der Behandlung einer psychischen Krankheit mit einzelnen Ratings (Blöcke 1 und 2) oder Angaben in Block 3 korrelierte^1^. Für Block 1 konnten positiv signifikante Zusammenhänge für die Items 1.1 (r = 0,232, *p* = 0,039; „erhöhte Belastung im Modul 5.1“), 2.3 (r = 0,261, *p* = 0,020; „Belastung durch das Fach psychosomatische Medizin“) und 3.2 (r = 0,225, *p* = 0,048; „Belastung durch Kontakt mit psychisch kranken Menschen im Seminar“) gefunden werden – diese sind nach Cohen (1988) als kleine Effekte zu interpretieren. Für Block 2 wurde für „Essstörung“ ein signifikant positiver Zusammenhang gefunden (r = 0,335, *p* = 0,003; mittlerer Effekt). Der Status einer psychischen Erkrankung korrelierte nicht signifikant mit den Items aus Block 3 (Wünsche nach/Hinweise auf Unterstützungsangebote).

## Diskussion

Ziel der vorliegenden Studie war die Objektivierung der emotionalen Belastung von Medizinstudierenden während der Lehre zu psychischen Krankheitsbildern. Bisher wurden lediglich das generelle Stresslevel und der hohe Anteil psychischer Erkrankungen bei Studierenden und Medizinstudierenden untersucht [2, 4, 7, 11, 16, 17] – diese Lücke sollte geschlossen werden. Unsere Studie konnte erstmals die hohe emotionale Belastung von Medizinstudierenden im Zusammenhang mit dem Erlernen psychischer Erkrankungen nachweisen. Die Belastung der Studierenden war hierbei insbesondere im Fach Psychiatrie erhöht sowie bei unmittelbarem Patientenkontakt und im Umgang mit an Depression und an PTBS erkrankten Patienten. Eine eigene psychische Vorerkrankung hing tendenziell mit höheren Belastungslevels zusammen. Fast die Hälfte der befragten Studierenden wünschte sich entsprechende Hinweise auf Unterstützungsangebote für den Fall der eigenen emotionalen Belastung.

Sowohl die hohe Rücklauf- (70,3 %) als auch Ausfüllquote (94,0 %) unterstreichen die Relevanz des Themas für die Studierenden. Die angegebenen Teilnahmequoten zu Vorlesungsbesuch (74,7 %) sowie Seminaren und UaK (95,2 %) waren plausibel, was eine gute Abbildung der befragten Studienpopulation impliziert.

Die Ergebnisse sprechen klar für eine erhöhte emotionale Belastung der Studierenden während der Lehre zu psychischen Erkrankungen: Die Studierenden gaben an, im Vergleich zur somatischen Lehre emotional belasteter zu sein (Zustimmung >6 von 10) – 43,2 % im Bereich der hohen Belastung (Werte von 8–10). Somit wurde nicht nur das aus der Literatur bekannte hohe Stresslevel bei Medizinstudierenden bestätigt [4, 11, 12, 16], sondern die Beschäftigung mit psychischen Erkrankungen als gesonderter Stressor identifiziert. Für diese These spricht, dass der Grad der emotionalen Belastung mit der Intensität des Patientenkontakts zunahm, mit höchster Ausprägung im UaK gegenüber den theoretischen Lehrveranstaltungen. Weiterhin war zum Zeitpunkt des UaKs die Modulklausur bereits geschrieben (siehe Abschn.: Beschreibung der Lehre: Modul 5.1) und fiel als relevanter Stressor des Medizinstudiums aus [13, 17].

Neben dem Grad des Patientenkontakts lassen sich Hinweise für die erhöhte Belastung in den Freitextantworten der Studierenden finden: Mehrfach genannt wurden intensive Schilderung von Schicksalsschlägen und Identifikation mit Patienten in ähnlichem Alter. Diese Altersgruppe kommt in Psychiatrie und Psychosomatik häufiger vor als in den meisten somatischen Fächern [3]. Hierzu passend wurde das Fach Kinder- und Jugendpsychiatrie als signifikant weniger emotional belastend bewertet.

Bezogen auf Krankheitsbilder wurde die Beschäftigung mit depressiven Störungen als am stärksten belastend bewertet – diese treten häufig erstmals im Alter der Studierenden auf [5] und erlauben somit eine größere Identifikation mit den Betroffenen. Umgekehrt wurde der Umgang mit Demenzen, die fast ausschließlich im hohen Lebensalter auftreten [6], als am wenigsten belastend angegeben.

Etwa die Hälfte der befragten Studierenden äußerte den Wunsch nach einer Anlaufstelle bei hoher eigener emotionaler Belastung. Neben dem Wunsch nach einer offenen Sprechstunde erreichten auch die anderen genannten Optionen hohe Zustimmung. Allerdings stellt die Inanspruchnahme von Hilfsangeboten bei psychischen Krisen für Medizinstudierende selbst eine emotionale Hürde dar – vor allem Sorgen vor Stigmatisierung oder fehlender Vertraulichkeit sind Gründe für eine Nichtinanspruchnahme [1, 8]. Es erscheint sinnvoll, Ansprechpartner nicht direkt aus dem Kreis der Lehrenden zu rekrutieren sowie Vertraulichkeit zu garantieren und dies gegenüber den Studierenden zu kommunizieren.

Knapp ein Fünftel der Studierenden (18,1 %) gab an, sich wegen einer psychischen Erkrankung in Behandlung zu befinden, was in etwa der Prävalenz in studentischen Populationen aus anderen Studien entspricht [7, 14, 18]. Tendenziell stieg mit diesem Status die emotionale Belastung durch die Lehre. Auch stimmten von den 15 betroffenen Studierenden drei der Aussage im hohen Bereich zu (Werte von 8–10), ihre Erkrankung habe sich während der Lehre zu psychischen Erkrankungen verschlechtert. Ein stärkerer Wunsch nach Unterstützungsangeboten wurde jedoch nicht geäußert, wobei bereits bestehende Behandlungsstrukturen hier eine mögliche Erklärung darstellen.

### Limitationen

(1) In der vorliegenden Studie wurde ein einzelnes Semester an einer Universität befragt. Ein multizentrischer Ansatz würde die Stichprobengröße sowie Repräsentativität bezogen auf die Population der Medizinstudierenden erhöhen. Auch die Analyse zusätzlicher Subgruppen (z. B. Differenzierung verschiedener somatischer Fächer) wäre so möglich. (2) Zwar kann davon ausgegangen werden, dass die studentische Population aus dem betrachteten Semester durch die hohe Teilnahmequote ausreichend abgebildet wurde, jedoch ist aufgrund der nicht erfassten Merkmale (z. B. Alter, Geschlecht) keine Analyse demographischer Subgruppen möglich. Gleichzeitig ist davon auszugehen, dass die Teilnahmequote bei Erfassung solcher Merkmale aufgrund von Sorgen über die eigene Identifizierbarkeit absinken würde. (3) In der hier vorliegenden Studie wurde ein selbst entwickelter Fragebogen zur Erfassung der subjektiven Belastung in der psychiatrischen Lehre entwickelt. Für den Fragebogen liegen bisher keine Daten zu dessen Reliabilität und Validität vor. In Zukunft sollte insbesondere die konvergente Validität des erfassten Konstrukts der psychischen Belastung durch Hinzuziehen etablierter Instrumente bestätigt werden. (4) Eine zentrale Evaluation, die das Belastungslevel in allen Fächern zeitnah nach der durchgeführten Lehre im Laufe des Medizinstudiums erfasst, würde einen direkten Vergleich zwischen den Fächern ermöglichen. Dies war in der hier durchgeführten Studie nicht umsetzbar, sollte aber zukünftig etabliert werden. (5) Die Ergebnisse unserer Studie sind, da an einer Universität mit Regelstudiengang gewonnen, nicht uneingeschränkt auf den Modellstudiengang Humanmedizin übertragbar. Allerdings sind auch in diesem ein psychiatrisches Blockpraktikum bzw. UaK vorgesehen, sodass eine vergleichbare emotionale Belastung zumindest im direkten Patientenkontakt zu erwarten ist.

## Zusammenfassung und Fazit für die Praxis

- Die universitäre Lehre zu psychischen Erkrankungen stellt für einen relevanten Teil der Medizinstudierenden einen emotional belastenden Abschnitt ihres Studiums dar. Die Belastung steigt mit der Intensität des Patientenkontakts und bei eigenen psychischen Vorerkrankungen. Gleichzeitig wird seitens der Studierenden der Bedarf nach Unterstützungsangeboten geäußert.
- Die potenzielle emotionale Belastung für Medizinstudierende sollte in der Planung von Lehrveranstaltungen zu psychischen Erkrankungen mitgedacht und beteiligte Lehrpersonen für dieses Thema sensibilisiert werden. Hinweise auf bestehende Angebote, wie z. B. psychosoziale Ambulanzen für Studierende, oder Triggerwarnungen stellen einfach umzusetzende Möglichkeiten der Unterstützung dar. Idealerweise wird im Vorfeld der Lehre eine nicht an der Lehre beteiligte Person benannt, die als Ansprechpartner für die Studierenden in emotionalen Krisen zur Verfügung steht.

## Data Availability

Die der Studie zugrundeliegenden Daten sind aus Datenschutzgründen nicht öffentlich verfügbar. Bei berechtigtem Interesse können die Daten bei den Autoren angefordert werden.

## Footnotes

1 Für die Korrelation mit metrischen Variablen wurden Pearson-Korrelationen berechnet. Für die Korrelation zwischen kategorischen Variablen wurden 4‑Felder-Phi-Koeffizienten berechnet.
